# Germline breast cancer susceptibility gene mutations and breast cancer outcomes

**DOI:** 10.1186/s12885-018-4229-5

**Published:** 2018-03-22

**Authors:** Yong Alison Wang, Jhih-Wei Jian, Chen-Fang Hung, Hung-Pin Peng, Chi-Fan Yang, Hung-Chun Skye Cheng, An-Suei Yang

**Affiliations:** 1 Department of Internal Medicine, Koo Foundation Sun-Yat Sen Cancer Center, Taipei, Taiwan; 2 Genomic Research Center, Academia Sinica, Taipei, Taiwan; 3 Institute of Biomedical Informatics, National Yang-Ming University, Taipei, Taiwan; 4 Bioinformatics Program, Taiwan International Graduate Program, Institute of Information Science, Academia Sinica, Taipei, Taiwan; 5 Department of Research, Koo Foundation Sun-Yat Sen Cancer Center, Taipei, Taiwan; 6 Institute of Biomedical Sciences, Academia Sinica, Taipei, Taiwan; 7 Department of Radiation Oncology, Koo Foundation Sun-Yat Sen Cancer Center, Taipei, Taiwan

**Keywords:** Cancer susceptibility gene, Breast cancer, *BRCA1* & *BRCA2*, Breast cancer prognosis, Next generation sequencing

## Abstract

**Background:**

It is unclear whether germline breast cancer susceptibility gene mutations affect breast cancer related outcomes. We wanted to evaluate mutation patterns in 20 breast cancer susceptibility genes and correlate the mutations with clinical characteristics to determine the effects of these germline mutations on breast cancer prognosis.

**Methods:**

The study cohort included 480 ethnic Chinese individuals in Taiwan with at least one of the six clinical risk factors for hereditary breast cancer: family history of breast or ovarian cancer, young age of onset for breast cancer, bilateral breast cancer, triple negative breast cancer, both breast and ovarian cancer, and male breast cancer. PCR-enriched amplicon-sequencing on a next generation sequencing platform was used to determine the germline DNA sequences of all exons and exon-flanking regions of the 20 genes. Protein-truncating variants were identified as pathogenic.

**Results:**

We detected a 13.5% carrier rate of pathogenic germline mutations, with *BRCA2* being the most prevalent and the non-*BRCA* genes accounting for 38.5% of the mutation carriers. *BRCA* mutation carriers were more likely to be diagnosed of breast cancer with lymph node involvement (66.7% vs 42.6%; *P* = 0.011), and had significantly worse breast cancer specific outcomes. The 5-year disease-free survival was 73.3% for *BRCA* mutation carriers and 91.1% for non-carriers (hazard ratio for recurrence or death 2.42, 95% CI 1.29–4.53; *P* = 0.013). After adjusting for clinical prognostic factors, *BRCA* mutation remained an independent poor prognostic factor for cancer recurrence or death (adjusted hazard ratio 3.04, 95% CI 1.40–6.58; *P* = 0.005). Non-*BRCA* gene mutation carriers did not exhibit any significant difference in cancer characteristics or outcomes compared to those without detected mutations. Among the risk factors for hereditary breast cancer, the odds of detecting a germline mutation increased significantly with having bilateral breast cancer (adjusted odds ratio 3.27, 95% CI 1.64–6.51; *P* = 0.0008) or having more than one risk factor (odds ratio 2.07, 95% CI 1.22–3.51; *P* = 0.007).

**Conclusions:**

Without prior knowledge of the mutation status, *BRCA* mutation carriers had more advanced breast cancer on initial diagnosis and worse cancer-related outcomes. Optimal approach to breast cancer treatment for *BRCA* mutation carriers warrants further investigation.

**Electronic supplementary material:**

The online version of this article (10.1186/s12885-018-4229-5) contains supplementary material, which is available to authorized users.

## Background

Multigene panel testing is increasingly adopted for managing breast cancer susceptibility in high risk individuals suspected of having hereditary breast cancer, but the evidence-based practice guidelines remain far from being comprehensive. The advent of next generation sequencing (NGS) technologies is making multigene panel testing easier and affordable [1–4]. In addition, multigene panel testing could identify up to 50% more individuals with cancer susceptibility gene mutations in comparison with testing only for *BRCA1* and *BRCA2* (*BRCA*) [5]. Most of these additional mutations are from moderate risk genes, many of which could result in alterations of cancer risk estimation and clinical action [5, 6]. However, arriving at consistent and optimal clinical recommendations on the basis of the interpretations of the multigene panel testing and associated variants of uncertain significance (VUS) could be challenging due to lack of comprehensive understanding on the consequences of the genetic alterations [7]. As the multigene panel testing is becoming widely adopted, studies are needed to develop evidence-based practice guidelines.

In addition to risk assessment of breast cancer susceptibility in germline mutation carriers, understanding prognosis after breast cancer diagnosis will also impact practice guidelines for breast cancer. With the efficacy of poly (ADP-ribose) polymerase (PARP) inhibitors in controlling *BRCA* mutation positive tumors, many clinical trials are now underway evaluating their use in breast cancer [8]; their incorporation into systemic therapy in clinical practice is highly anticipated. It has not been established whether *BRCA* or any cancer susceptibility gene mutation is an independent prognostic factor after breast cancer diagnosis. Despite the suspicion for a more aggressive tumor phenotype, most studies have fallen short of showing differences in clinical outcomes in *BRCA* mutation carriers [9–12]. Systematic reviews with larger pooled sample size have yielded conflicting conclusions, possibly due to variability of included studies [13–15]. Consequently, conventional decisions regarding systemic therapy for *BRCA* mutation-associated breast cancer have been based on disease characteristics rather than *BRCA* mutation status. As such, it would be informative to discover causal or statistical correlations of germline breast cancer susceptibility gene mutations to breast cancer prognosis.

A panel of 20 known and candidate breast cancer susceptibility genes were selected herein for the multigene panel testing study. Among the 20 genes, *BRCA1, BRCA2, PALB2, TP53, CDH1, PTEN, ATM, CHEK2, BARD1, STK11, NBN* have been well established as breast cancer susceptibility genes [7, 16, 17]. Some are part of rare high-penetrance cancer predisposing syndromes (e.g. *BRCA1, BRCA2, TP53, CDH1, PTEN, STK11, PALB2*) while others are moderate-penetrance genes (e.g. *ATM, NBN, CHEK2, BARD1)*. The impacts of the mutations in *RAD50*, *RAD51C*, and *RAD51D* on breast cancer susceptibility and survival are controversial: Mutations in *RAD50* have been found not associated with breast cancer risk [17]. Also mutations in *RAD51C* have not been found to increase the risk of breast cancer [18–20] and mutations in *RAD51D* have been associated with high risk of ovarian cancer but not with breast cancer [21]. Nevertheless, other studies have indicated that mutations in *RAD51C* [22] and *RAD51D* [17, 23, 24] contribute to the risk of both breast and ovarian cancer, and that *RAD50* is an intermediate-risk breast cancer susceptibility gene [25]. Although germline mutations in the DNA mismatch repair genes (*MLH1, MSH2, MSH6, PMS2*) have been mostly associated with Lynch syndrome, evidence has been established to support the connections between the mutations in the DNA mismatch repair genes and the risk or survival of breast cancer [26–29]. Similarly, whether *BRIP1* is a breast cancer susceptibility gene remains controversial [30], and perhaps is dependent on the ethnicity of the cohort studied [22]. *NF1* mutations have been known to associate with increased risk of breast cancer in younger population [31] and poor breast cancer survival [32]. To clarify the controversies, we included in the panel the potentially relevant genes above to explore the germline mutation-dependence of breast cancer predisposition and outcomes in our local high risk population.

Different ethnic populations need respective studies on cancer risks pertinent to germline mutations. In the western populations, about 5% of the breast cancer patients may carry heritable cancer susceptibility gene mutations [33, 34]. *BRCA1* and *BRCA2* account for the majority of these gene mutations, with *BRCA1* being the most common [34, 35]. However, studies in Asian populations have indicated somewhat different conclusions: available results show that *BRCA* mutation rates in Asians are lower than those in Whites, and that the distributions of the gene mutations are also different [36–43]. It is imperative to enrich mutation databases on different ethnic populations, so as to better interpret ethnically specific germline mutations and better manage cancer risks among corresponding ethnic populations.

In this study, we analyzed germline mutations in the 20 breast cancer susceptibility genes using NGS-based technique in a cohort of high risk ethnic Chinese population. We evaluated the correlation of mutations with clinical characteristics and cancer outcomes. We aimed to clarify the prognostic value of *BRCA* and other breast cancer susceptibility gene mutations on breast cancer specific outcomes after conventional cancer treatment.

## Methods

### Study participants and data collection

Koo Foundation Sun Yat-Sen Cancer Center (KF-SYSCC) treats over 1000 newly diagnosed breast cancer patients annually. Between July 30, 2015 and March 31, 2016, we enrolled 480 individuals fulfilling at least one of the six eligibility criteria: family history of breast or ovarian cancer at any age (2 or more individuals on the same lineage of the family), personal history of breast cancer with age of diagnosis less than or equal to 40, bilateral breast cancer diagnosed at the same time or sequentially, triple negative (ER/PR/HER2 negative) breast cancer, breast and ovarian cancer in the same individual, and male breast cancer. None of the participants had known mutation status in any cancer susceptibility genes prior to enrollment. Clinical information was collected through participant surveys, electronic medical records, and the institutional breast cancer database**.**

### Sequencing and variant analyses of cancer susceptibility genes in genomic DNA

Germline DNA sequencing of all exonal regions was done in twenty breast cancer susceptibility genes: *BRCA1, BRCA2, PTEN, TP53, CDH1, STK11, NF1, NBN, MLH1, MSH2, MSH6, PMS2, ATM, BRIP1, CHEK2, PALB2, RAD50, BARD1, RAD51C*, and *RAD51D*. Polymerase chain reaction (PCR)-enriched amplicon-sequencing on an NGS platform was used to sequence genomic DNA extracted from whole blood or frozen buffy coat samples using the Gentra Puregene Blood kit (Qiagen, Minneapolis, MN, USA). The DNA samples were first PCR amplified using the Qiagen GeneRead DNAseq custom panel primer sets for the 20 genes, covering all exons as well as at least 10-base exon-flanking regions. The Qiagen primer set included 1184 amplicons and provided at least 90% coverage for most genes except for *STK11* (59%), *PMS2* (74%) and *MSH2* (89%). PCR enriched amplicons were end-repaired, adenylated, and ligated to NEXTflex-96 DNA barcodes (Bioo Scientific, Austin, Texas, USA) using the Qiagen GeneRead DNA Library I Core Kit. Barcoded libraries were amplified using the Qiagen GeneRead DNA I Amp Kit and NEXTflex primers (Bioo Scientific). Quality control and quantification of libraries were performed using the Qubit dsDNA HS Assay kit and the Agilent DNA 1000 kit. The barcoded DNA libraries were pooled in equal amounts and underwent 2x150bp paired-end sequencing on an Illumina MiSeq platform. The average base call error rates were less than 1.0%.

We constructed a pipeline based on public domain software and databases for alignment, variant calling, and annotation, using GRCh37 as the reference genome. BWA (http://bio-bwa.sourceforge.net/) was used to map reads to the reference genome. Bam-readcount (https://github.com/genome/bam-readcount) was used to count variants for each aligned position. Variant calling protocols were carried out either based on GATK Best Practices (https://software.broadinstitute.org/gatk/best-practices/) or using a non-GATK based algorithm, where lower limits of 50 for read depth and 10% for proportion of raw reads with a variant were used for variant calling. Variants that were intergenic, intronic (except for the 10 bp exon-flanking regions), or synonymous (sense) were excluded. All other variants identified with the two algorithms were compared, and discrepant variants were manually inspected by viewing the BAM reads using the Integrative Genomics Viewer (IGV, Broad Institute, Inc.) to decide on the validity of the variant. The variants were searched in the dbSNP database (http://www.ncbi.nlm.nih.gov/SNP/) and the ClinVar database (http://www.ncbi.nlm.nih.gov/clinvar/). Variants were annotated as pathogenic, uncertain significance, or benign, using variant-dependent methods and disease-dependent methods. Nonsense, frameshift, and splice-site mutations that result in a truncated protein product were classified as pathogenic. The clinical significance interpretation on ClinVar, if available, was referenced for categorization. Novel missense mutations not found in the public databases were classified as variants of uncertain significance. We used various *in silico* models (Align-GVGD [44], PolyPhen-2 [45], SIFT [46], PROVEAN [47], CADD [48]) to evaluate the deleteriousness of the variants, especially missense variants. However, we did not change classification based on the *in silico* models. As the interpretation of missense variants is often controversial [7], we took a more conservative approach of only including the protein-truncating variants in the clinical correlation of this study. All variants classified as pathogenic were further verified using the Sanger sequencing method, confirming they were germline mutations.

### Detection of large genomic rearrangement using copy number variation (CNV) analyses

Coverage or read depth has been used to detect CNVs in genome-scale (whole genome sequencing) datasets. Multiplex PCR-based enrichment focuses sequencing efforts on a very small fraction of the genome, and the observed read depth for each of the regions can differ due to varying number of PCR amplicons, sequence variation, or PCR enrichment efficiency. For CNV detection in our PCR-enriched amplicon sequencing data of the 20 genes, we used two algorithms, Quandico [49] and ONCOCNV [50], specifically developed for CNV analysis of amplicon sequencing data. The CNVs detected with these algorithms were then verified experimentally using the multiplex ligation-dependent probe amplification (MLPA) technique. This analysis resulted in the discovery of two carriers of a *BRCA1* large genomic rearrangement (LGR) in the study cohort.

### Clinical correlation and statistical analyses

For study participants who have had breast cancer, tumor characteristics and clinical outcomes were extracted from the institutional breast cancer database and participant survey. Correlation statistics between clinical characteristics and *BRCA* mutation or non-*BRCA* mutation status were performed using the Chi-square test or t test.

For the correlation analyses of clinical outcomes and germline mutations, we performed survival analyses using Kaplan-Meier curves and Cox proportional hazards regression analysis. The primary end point was disease-free survival, defined as the time from breast cancer surgery to the first appearance of one of the following: invasive recurrence of breast cancer (local, regional, or distant) or death without breast-cancer recurrence. Secondary end points included the following: time interval without breast cancer recurrence, defined as the time from breast cancer surgery to the recurrence of invasive breast cancer (local, regional, or distant); time interval before a recurrence of breast cancer at a distant site, defined as the time from breast cancer surgery to the recurrence of breast cancer at a distant site; and overall survival, defined as the time from breast cancer surgery to death from any cause. For patients who did not have an end-point event, the times were censored at the date of the last follow-up visit (or for the analysis of overall survival, the date at which the patient was last known to be alive).

The above primary and secondary end points of the groups with different mutation status were compared using Kaplan-Meier curves, and the statistical significance was evaluated using the log-rank test. Cox proportional hazards regression analysis was used to evaluate univariate and multivariate hazard ratios (HR) for *BRCA* germline mutation for the end points. The covariates for the multivariate analyses included: tumor size > 2 cm, lymph node positivity, triple negative tumor type, young age of onset (≤ 40), mastectomy (vs breast-conserving surgery), adjuvant chemotherapy, adjuvant radiotherapy, and hormonal therapy.

The six eligibility criteria for study enrollment were considered clinical risk factors for having a germline mutation in the cancer susceptibility genes sequenced. The odds ratios (OR) of having a pathogenic germline mutation were calculated using multivariable logistic regression with the six dichotomous risk factors as independent variables, and having a pathogenic germline mutation as the dependent variable. The odds ratios for the number of risk factors were obtained by logistic regression. Statistical significance was represented as 95% confidence intervals and *P*-values. An alpha level of 0.05 was defined as statistically significant for rejecting the null hypothesis. All analyses were performed using SAS 9.4® (SAS Institute, Cory, NC, USA).

## Results

### Study population

All 480 individuals in the study cohort were ethnically Chinese. In the cohort, 95.4% (458 individuals) had a personal history of breast cancer. The mean age of onset for breast cancer was 41.8 (range 17–82). The proportions (numbers) of individuals with each risk factor were: family history 47.5% (228), age of onset ≤40 54.2% (260), bilateral breast cancer 11.3% (54), triple-negative breast cancer 26.5% (127), breast and ovarian cancer in the same woman 0.8% (4), and male breast cancer 1.3% (6).

### Characteristics of the germline mutations

**Fig. 1:**
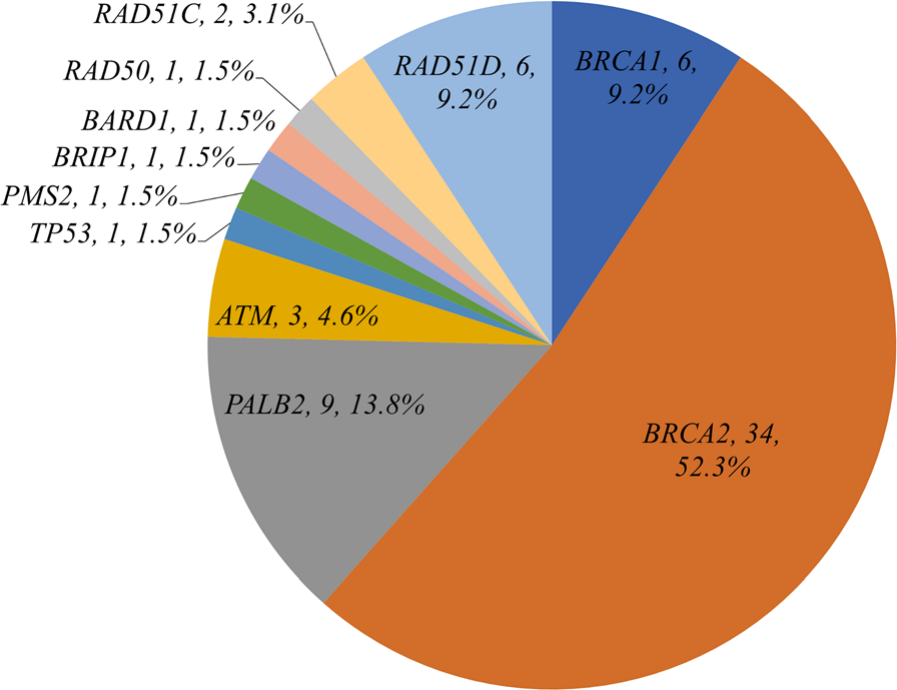
Distribution of the 20 breast cancer susceptibility genes. Genes not shown (*PTEN, CDH1, STK11, NF1, NBN, MLH1, MSH2, MSH6, CHEK2*) are those without identified pathogenic mutations in the study cohort

To survey the breast cancer susceptibility gene mutations, we sequenced the panel of 20 genes from all individuals in the study cohort. We identified 47 pathogenic mutations carried in 65 individuals - a detection rate of 13.5% in 11 genes and 8.3% (40) in *BRCA* genes in this high risk population. Pathogenic mutations in *BRCA2* were the most prevalent, comprising 52.3% (34) of the 65 pathogenic mutation carriers (Fig. 1); individuals with *BRCA1* mutations were only 9.2% (6). *PALB2* was the second most common gene to have pathogenic mutations with 13.8% (9) carrier rate. Non-*BRCA* gene mutations contributed to 38.5% (25) - a significant portion of the pathogenic mutation carriers.

A substantial portion of the pathogenic mutations are novel. Table 1 summarizes the 47 identified pathogenic mutations, which contain 15 nonsense, 28 frameshift, 3 splice-site variants, and 1 large genomic rearrangement (multiple exon deletion). Seventeen (36.2%) of the pathogenic mutations have not been recorded in the dbSNP database, designated as novel variants. We also identified 173 variants of uncertain significance (VUS), of which 45 (26.0%) were novel; 183 (38.1%) in the study cohort carried at least one VUS.

**Table 1:** Pathogenic germline mutations detected in the 20 breast cancer susceptibility genes in a high risk population for hereditary breast cancer in Taiwan (*N* = 480)

Gene	HGVS notation	Type of variant	No. of carriers	SNP ID
*BRCA1*	NM_007294.3:c.5075-1G > A	Splice site	1	rs1800747
*BRCA1*	NM_007294.3:c.4678_4679delGG (p.Gly1560Asnfs)	Frameshift	2	Novel
*BRCA1*	NM_007294.3:c.3644_3648delACTTA (p.Asn1215Ilefs)	Frameshift	1	Novel
*BRCA1*	deletion of exon 1 to 16	LGR	2	Novel
*BRCA2*	NM_000059.3:c.-7_9del16	Frameshift	1	Novel
*BRCA2*	NM_000059.3:c.469_470delAA (p.Lys157Valfs)	Frameshift	2	rs397507739
*BRCA2*	NM_000059.3:c.755_758delACAG (p.Asp252Valfs)	Frameshift	1	rs80359659
*BRCA2*	NM_000059.3:c.799dupG (p.Thr269Asnfs)	Frameshift	1	Novel
*BRCA2*	NM_000059.3:c.857C > G (p.Ser286Ter)	Nonsense	1	Novel
*BRCA2*	NM_000059.3:c.2095C > T (p.Gln699Ter)	Nonsense	1	rs878853559
*BRCA2*	NM_000059.3:c.2442delC (p.Met815Trpfs)	Frameshift	1	rs397507627
*BRCA2*	NM_000059.3:c.2754delC (p.Asn918Lysfs)	Frameshift	1	Novel
*BRCA2*	NM_000059.3:c.2808_2811delACAA (p.Ala938Profs)	Frameshift	1	rs80359351
*BRCA2*	NM_000059.3:c.2990 T > G (p.Leu997Ter)	Nonsense	1	Novel
*BRCA2*	NM_000059.3:c.3109C > T (p.Gln1037Ter)	Nonsense	3	rs80358557
*BRCA2*	NM_000059.3:c.3322A > T (p.Lys1108Ter)	Nonsense	1	Novel
*BRCA2*	NM_000059.3:c.3883C > T (p.Gln1295Ter)	Nonsense	1	rs879255309
*BRCA2*	NM_000059.3:c.4914dupA (p.Val1639Serfs)	Frameshift	1	rs786203494
*BRCA2*	NM_000059.3:c.5141_5144delATTT (p.Tyr1714Cysfs)	Frameshift	1	rs80359487
*BRCA2*	NM_000059.3:c.5164_5165delAG (p.Ser1722Tyrfs)	Frameshift	6	rs80359490
*BRCA2*	NM_000059.3:c.5621_5624delTTAA (p.Ile1874Argfs)	Frameshift	1	rs80359526
*BRCA2*	NM_000059.3:c.6275_6276delTT (p.Leu2092Profs)	Frameshift	1	rs11571658
*BRCA2*	NM_000059.3:c.6490C > T (p.Gln2164Ter)	Nonsense	1	rs397507860
*BRCA2*	NM_000059.3:c.6800C > A (p.Ser2267Ter)	Nonsense	1	rs377698594
*BRCA2*	NM_000059.3:c.8203delC (p.Leu2736Serfs)	Frameshift	1	Novel
*BRCA2*	NM_000059.3:c.8234dupT (p.Thr2746Aspfs)	Frameshift	1	rs276174903
*BRCA2*	NM_000059.3:c.8400_8402delTTTinsAAAA (p.Phe2801Lysfs)	Frameshift	1	rs483353077
*BRCA2*	NM_000059.3:c.8485C > T (p.Gln2829Ter)	Nonsense	1	rs80359099
*BRCA2*	NM_000059.3:c.8961_8964delGAGT (p.Ser2988Phefs)	Frameshift	1	rs80359734
*BRCA2*	NM_000059.3:c.9227delG (p.Gly3076Aspfs)	Frameshift	1	rs397508040
*PALB2*	NM_024675.3:c.3143delA (p.Lys1048Argfs)	Frameshift	1	Novel
*PALB2*	NM_024675.3:c.2968G > T (p.Glu990Ter)	Nonsense	1	rs876659036
*PALB2*	NM_024675.3:c.2480_2481delCA (p.Thr827Metfs)	Frameshift	1	Novel
*PALB2*	NM_024675.3:c.2257C > T (p.Arg753Ter)	Nonsense	1	rs180177110
*PALB2*	NM_024675.3:c.1059delA (p.Lys353Asnfs)	Frameshift	1	rs730881872
*PALB2*	NM_024675.3:c.1050_1051delAAinsTCT (p.Gln350Hisfs)	Frameshift	2	rs180177098
*PALB2*	NM_024675.3:c.643G > T (p.Glu215Ter)	Nonsense	2	Novel
*ATM*	NM_000051.3:c.2284_2285delCT (p.Leu762Valfs)	Frameshift	2	rs587781658
*ATM*	NM_000051.3:c.6312G > A (p.Trp2104Ter)	Nonsense	1	Novel
*TP53*	NM_000546.5:c.416_420dupAGACC (p.Cys141Argfs)	Frameshift	1	Novel
*PMS2*	NM_000535.5:c.1144 + 1G > A	Splice site	1	rs373885654
*BRIP1*	NM_032043.2:c.2244C > G (p.Tyr748Ter)	Nonsense	1	Novel
*BARD1*	NM_000465.3:c.654G > A (p.Trp218Ter)	Nonsense	1	Novel
*RAD50*	NM_005732.3:c.2157dupA (p.Glu723Glyfs)	Frameshift	1	rs397507178
*RAD51C*	NM_058216.2:c.394dupA (p.Thr132Asnfs)	Frameshift	1	rs730881940
*RAD51C*	NM_058216.2:c.905-2A > C	Splice site	1	rs779582317
*RAD51D*	NM_002878.3:c.270_271dupTA (p.Lys91Ilefs)	Frameshift	6	rs753862052

*LGR*: large genomic rearrangement

### Correlation of mutations with tumor characteristics

To characterize the clinical feature upon cancer diagnosis in the germline mutation carriers, we compared clinical characteristics among 3 subgroups of the cohort with different mutation status. Among the 457 patients with breast cancer characteristics and clinical outcomes available, the tumor characteristics of those with *BRCA* mutation or with non-*BRCA* mutation are compared with those without any detected pathogenic germline mutation in Table 2A. None of the patients had prior knowledge of cancer susceptibility gene mutation status at the time of their breast cancer diagnosis or treatment. Those carrying *BRCA* mutations were more likely to have lymph node involvement upon initial breast cancer diagnosis when compared with those without mutations (66.7% vs 42.6%; *P* = 0.011). Probably as a result, more *BRCA* mutation carriers received chemotherapy (100% vs 82%; *P* = 0.005) and had mastectomy as opposed to breast conserving surgery (80.6% vs 62.1%; *P* = 0.028). Other cancer characteristics, including age of onset, tumor size, overall stage, estrogen receptor, HER2 overexpression, nuclear grade, lymphovascular invasion, receipt of radiotherapy or hormonal therapy, did not show statistically significant difference in the *BRCA* mutation carriers, as compared with those without mutations. In the non-*BRCA* mutation carrying breast cancer patients, all tumor characteristics were statistically indistinguishable from those of the breast cancer patients without germline mutations.

**Table 2:** Clinical characteristics (A) and outcomes (B) in the cohort of breast cancer patients (*N* = 457) and their correlation with *BRCA* mutation and non-*BRCA* mutation carrying status

		No mutation	*BRCA* mutation		Non-*BRCA* mutation	
		*N* = 397	*N* = 36	*P*-value*	*N* = 24	*P*-value*
*A. Clinical characteristics*						
Age of onset, mean (SD)		41.7 (9.9)	42.1 (10.1)	0.846	42.3 (11.4)	0.771
		*no. (%)*	*no. (%)*		*no. (%)*	
Lymph node	positive	160 (42.6)	20 (66.7)	**0.011**	8 (36.4)	0.568
	negative	216 (57.5)	10 (33.3)		14 (63.6)	
Tumor size	≤ 2 cm	194 (52.3)	16(55.2)	0.765	13 (59.1)	0.535
	> 2 cm	177 (47.7)	13 (44.8)		9 (40.9)	
Surgery type	MRM	246 (62.1)	29 (80.6)	**0.028**	14 (58.3)	0.711
	BCT	150 (37.9)	7 (19.4)		10 (41.7)	
Chemotherapy	yes	323 (82.0)	36 (100)	**0.005**	17 (70.8)	0.174
	no	71 (18.0)	0 (0)		7 (29.2)	
Radiotherapy	yes	275 (69.8)	29 (82.9)	0.103	16 (66.7)	0.746
	no	119 (30.2)	6 (17.1)		8 (33.3)	
Hormonal therapy	yes	217 (57.7)	21 (70)	0.189	12 (54.5)	0.770
	no	159 (42.3)	9 (30)		10 (45.5)	
Stage	0 (DCIS)	11 (2.8)	0 (0)	0.307	1 (4.2)	0.704
	1	138 (35.0)	8 (23.5)		11 (45.8)	
	2	159 (40.4)	15 (44.1)		10 (41.7)	
	3	48 (12.2)	8 (23.5)		1 (4.2)	
	4	7 (1.8)	0 (0)		0 (0)	
	LABC	31 (7.9)	3 (8.8)		1 (4.2)	
Triple negative	yes	108 (27.2)	9 (25.0)	0.776	9 (37.5)	0.274
	no	289 (72.8)	27 (75.0)		15 (62.5)	
ER	positive	234 (59.5)	24 (66.7)	0.403	12 (52.2)	0.485
	negative	159 (40.5)	12 (33.3)		11 (47.8)	
HER2 overexpression	yes	72 (19.8)	3 (8.8)	0.118	3 (14.3)	0.537
	no	292 (80.2)	31 (91.2)		18 (85.7)	
Nuclear grade	1	51 (13.9)	1 (3.5)	0.276	3 (14.3)	0.896
	2	123 (33.5)	11 (37.9)		8 (38.1)	
	3	193 (52.6)	17 (58.6)		10 (47.6)	
Lymphovascular invasion	prominent	82 (22.1)	11 (37.9)	0.122	2 (9.5)	0.259
	focal	104 (28.0)	8 (27.6)		5 (23.8)	
	absent	185 (49.9)	10 (34.5)		14 (66.7)	
*B. Outcomes*						
Recurrence†		53 (13.4)	11 (30.6)	**0.005**	1 (4.2)	0.191
Distant metastasis		38 (9.6)	9 (25.0)	**0.004**	0 (0)	0.112
Locoregional recurrence only		15 (3.8)	2 (5.6)	0.599	1 (4.2)	0.923
Death		12 (3.0)	3 (8.3)	0.095	0 (0)	0.388

*SD*: standard deviation; *MRM*: modified radical mastectomy; *BCT*: breast conserving therapy; *DCIS*: ductal carcinoma in situ; *LABC*: locally advanced breast cancer; *ER*: estrogen receptor; *HER2*: human epidermal growth factor receptor 2. * *P*-values were calculated using Chi-square test for categorical variables and t-test for continuous variables (age). The statistically significant values (< 0.05) are shown in bold. † Recurrence includes distant metastasis, local/ipsilateral breast and regional recurrence, and does not include contralateral breast cancer or second primary cancer

### Clinical outcomes

**Fig. 2:**
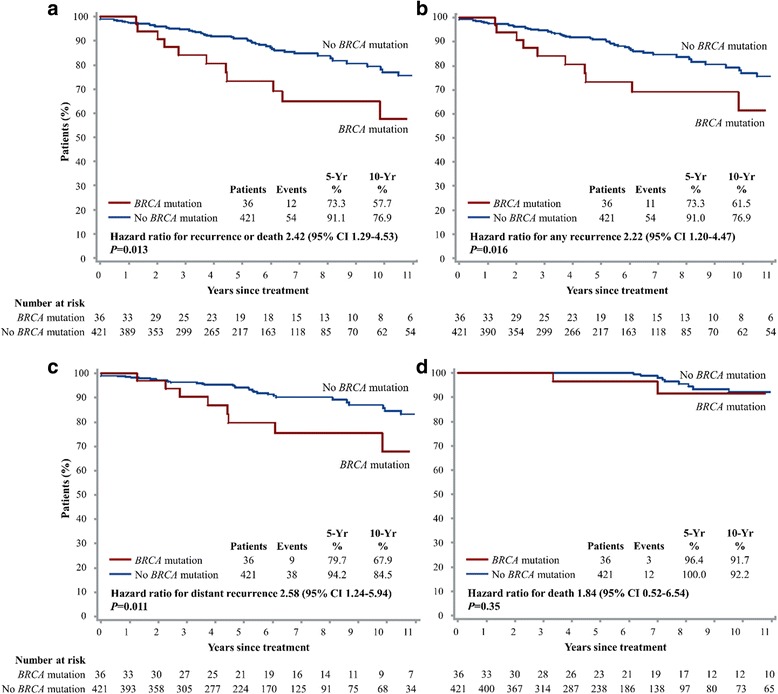
Kaplan-Meier estimates of disease-free survival (**a**), freedom from breast cancer recurrence (**b**), freedom from distant recurrence of breast cancer (**c**), and overall survival (**d**), according to *BRCA* mutation carrier status. The 5-year and 10-year values are based on Kaplan–Meier estimates of the time to an event. The hazard ratios are for breast cancer recurrence or death (**a**), any recurrence (**b**), distant recurrence (**c**), and death from any cause (**d**), respectively, based on Cox proportional hazards univariate analysis

To evaluate the prognostic value of *BRCA* germline mutation in breast cancer patients, we performed survival analyses comparing carriers and non-carriers of *BRCA* germline mutation for various clinical end points (Fig. 2). After a median follow-up of 66.9 months, 66 patients (14.4%) had breast cancer recurrence or died. The rate of disease-free survival at 5 years was 73.3% among *BRCA* mutation carriers, as compared with 91.1% among non-carriers (hazard ratio for recurrence or death 2.42, 95% CI 1.29–4.53; *P* = 0.013) (Fig. 2a). At 5 years, 73.3% of the *BRCA* mutation carriers were free from any breast cancer recurrence, as compared with 91.0% of the non-carriers (hazard ratio for recurrence 2.22, 95% CI 1.20–4.47; *P* = 0.016) (Fig. 2b). Distant recurrence of breast cancer was reported in 47 patients (10.3%), and at 5 years, 79.7% of the *BRCA* mutation carriers were free from distant metastasis, as compared with 94.2% of the non-carriers (hazard ratio for distant recurrence 2.58, 95% CI 1.24–5.94; *P* = 0.011) (Fig. 2c). Death was reported in 15 patients (3.3%); 1 patient died without breast cancer recurrence. Overall survival at 5 years was 96.4% among *BRCA* mutation carriers, as compared with 100% among non-carriers (hazard ratio for death 1.84, 95% CI 0.52–6.54; *P* = 0.35) (Fig. 2d). Survival analyses comparing *BRCA1* and *BRCA2* separately showed that *BRCA1* mutation carriers appeared to have the worst outcomes in the cohort, followed by *BRCA2* mutation carriers (Additional file 1: Fig. S1). The *BRCA* mutation carriers had significantly poorer breast cancer specific outcomes, most significantly in distant recurrence.

In the multivariate Cox proportional hazards model, *BRCA* mutation remained an independent poor prognostic factor for all cancer outcomes after adjustment for covariates including tumor size, lymph node status, triple negative tumor type, age, and treatment modalities (Table 3). The adjusted hazard ratios for *BRCA* mutation carriers were 3.04 (95% CI 1.40–6.58; *P* = 0.005) for recurrence or death, 2.70 (95% CI 1.20–6.06; *P* = 0.016) for any recurrence, 2.86 (95% CI 1.11–7.35; *P* = 0.029) for distant recurrence, and 8.01 (95% CI 1.44–44.7; *P* = 0.018) for any death. Among the clinical covariates, multivariate analysis showed poorer prognosis in large tumors, and favorable prognosis for those having had chemotherapy or with triple negative tumor types (Additional file 2: Table S2).

**Table 3:** Effects of *BRCA* germline mutation on breast cancer end points by multivariate Cox proportional hazards analysis

End Point	Adjusted Hazard Ratio* (95% CI)	*P*-value
Breast cancer recurrence or death	3.04 (1.40–6.58)	0.005
Locoregional or distant recurrence	2.70 (1.20–6.06)	0.016
Distant recurrence	2.86 (1.11–7.35)	0.029
Death	8.01 (1.44–44.7)	0.018

*The hazard ratios were calculated using multivariate Cox proportional hazards model adjusted for tumor size, lymph node status, triple negative status, age of breast cancer onset, surgery type, chemotherapy, radiotherapy, and hormonal therapy

To evaluate the prognostic consequences of the non-*BRCA* gene mutations, we compared the clinical outcomes between breast cancer patients with *BRCA* mutation, with non-*BRCA* mutation, and without mutation (Table 2B). The *BRCA* mutation carriers had a higher rate of any recurrence (30.6% vs 13.4%; *P* = 0.005) or distant recurrence (25.0% vs 9.6%; *P* = 0.004) than those with no mutation. In contrast, the non-*BRCA* mutation group had no distant metastasis or death and only one locoregional recurrence. No statistically significant difference in outcome was detected in this group as compared with those with no mutation. *PALB2* mutation was the most prevalent in the non-*BRCA* mutation group, and survival analyses comparing *PALB2* to *BRCA1*, *BRCA2* mutation carriers and to the remaining patients showed that *PALB2* mutation carriers had favorable prognosis (Additional file 1: Figure S1).

### Significance of risk factors for breast cancer susceptibility gene mutation

Correlations between the hereditary breast cancer risk factors and having a detectable germline mutation in any of the 20 genes were shown in Table 4. Multivariate analysis showed that the only significant risk factor in this high risk cohort was having bilateral breast cancer (synchronous or metachronous), with an adjusted odds ratio of 3.27 (95% CI 1.64–6.51; *P* = 0.0008). Family history, age of onset for breast cancer, and triple-negative breast cancer did not show significant correlation with the presence of mutation. The number of individuals with both breast and ovarian cancer or with male breast cancer was too small to detect any statistical significance.

**Table 4:** Correlation between clinical risk factors of hereditary breast cancer and having pathogenic germline mutations in the 20 breast cancer susceptibility genes

Risk factors		Mutation rate	OR (95% CI)*	*P*-value
Family history†	Yes	16.2% (37/228)	1.60 (0.90–2.88)	0.113
	No	11.1% (28/252)		
Age of onset	≤ 40	12.7% (33/260)	1.26 (0.69–2.31)	0.452
	> 40	13.6% (27/198)		
Triple negative breast cancer	Yes	14.2% (18/127)	1.27 (0.67–2.42)	0.469
	No	13.3% (47/353)		
Bilateral breast cancer	Yes	29.6% (16/54)	3.27 (1.64–6.51)	**0.0008**
	No	11.5% (49/426)		
Breast and ovarian cancer	Yes	50% (2/4)	5.90 (0.70–49.6)	0.103
	No	13.0% (63/476)		
Male breast cancer	Yes	16.7% (1/6)	2.35 (0.25–21.9)	0.454
	No	13.1% (64/474)		
No. of risk factors	1	10.4% (32/309)	1.82 (1.25–2.64)	**0.002**
	2	18.4% (27/147)		
	3	19.1% (4/21)		
	4	50% (1/2)		
	5	100% (1/1)		
All subjects		13.5% (65/480)		

* The adjusted odds ratios (OR) of having a pathogenic mutation in the 20 genes were calculated using multivariable logistic regression with the six dichotomous risk factors; the odds ratio for the no. of risk factors was obtained using univariate logistic regression. The statistically significant P-values (< 0.05) are shown in bold. † The presence of family history was defined as two or more persons on the same lineage of the family having breast or ovarian cancer. If the study participant had breast cancer, only one family member with breast/ovarian cancer was needed to qualify as having family history

Having higher number of risk factors in an individual was also significantly correlated with having a germline mutation. For each additional risk factor, the odds ratio of having a mutation was 1.82 (95% CI 1.25–2.64; *P* = 0.002). Having more than one risk factor was associated with an odds ratio of 2.07 (95% CI 1.22–3.51; *P* = 0.007) of detecting a mutation.

To focus on the impact of high penetrance breast cancer susceptibility genes, we repeated the correlation analysis for four high penetrance genes (*BRCA1, BRCA2, PALB2, TP53*), and for the two most prominent breast cancer susceptibility genes (*BRCA1*, *BRCA2*) (Additional file 3: Table S3). The results shown in Additional file 3: Table S3 did not lead to conclusions disagreeing with those based on the 20-gene analysis shown in Table 4, maintaining similar statistical correlations of having germline mutations with bilateral breast cancer and with more clinical risk factors.

## Discussion

We showed that *BRCA* germline mutation carriers in this large ethnic Chinese cohort were more likely to be diagnosed with breast cancer already spread to regional lymph nodes, and their breast cancer related outcomes were significantly worse. The 5-year disease-free survival rate was only 73.3% for *BRCA* mutation carriers, in contrast to 91.1% for non-mutation carriers. The *BRCA* mutation status was an independent prognostic factor with an adjusted hazard ratio of 3.04 (95% CI 1.40–6.58) for cancer recurrence or death. The poor clinical outcome in *BRCA* mutation carriers mainly resulted from recurrence as distant metastasis, therefore excluding the contribution by new primary cancer in the ipsilateral or contralateral breast, of which the risk had been known to be elevated in *BRCA* mutation carriers. Our result implied the more aggressive nature of breast tumors in *BRCA* germline mutation carriers. Most previous studies on clinical outcomes of *BRCA* mutation carriers have failed to show a significant prognostic effect by *BRCA* mutation [9–11, 13, 14]. However, a recent systematic review showed that both *BRCA1* and *BRCA2* mutation carriers had significantly worse breast cancer specific survival [15]. The discrepancy in these studies may in part result from limitations due to small sample sizes, lack of adjusting for disease characteristics, variations in mutation assay techniques, mutation types, cancer treatment modalities, or lengths of follow-up. Our study was conducted in an all-Chinese cohort where all study participants underwent the same NGS-based complete sequencing of the coding regions of *BRCA* genes among other genes. The majority of the mutations were in the *BRCA2* gene. The follow-ups were extensive in terms of length, with median duration over 5 years, and completeness. The tumor characteristics and outcome data had been collected in a prospective manner in a breast cancer registry as well as after the participants were enrolled. The majority of the breast cancer patients in the cohort (94%) had undergone treatment in a single cancer center where cancer characteristic-based treatment guideline was consistently adhered to. The homogeneity on data collection may have strengthened the validity of the prognostic analysis.

Studies have shown that tumor cells with *BRCA* mutations may show different response to different chemotherapy agents; they may have enhanced sensitivity to platinum while more resistant to taxanes. However, clinical studies on comparison of chemotherapy regimens in the *BRCA* mutation populations are limited [12]. For the breast cancer patients in our cohort, less than 10% received cisplatin in the neoadjuvant setting and none in the adjuvant setting, while about a third received a taxane (docetaxel) in the adjuvant setting. There were no significant differences in chemotherapy choice between the groups with and without *BRCA* mutation. Despite a higher rate of *BRCA* mutation carriers receiving chemotherapy, they had poorer cancer outcomes. Further prospective studies are needed to determine the optimal chemotherapy for *BRCA* mutation carriers. With the anticipated efficacy of incorporation of PARP inhibitors in the treatment of *BRCA* mutation associated breast tumors, knowledge of *BRCA* mutation status prior to initial cancer treatment becomes even more crucial.

In this Chinese cohort of high risk individuals for hereditary breast cancer, we found an overall prevalence of 13.5% for carriers of germline mutations in 11 of the 20 breast cancer susceptibility genes. In contrast to western populations, *BRCA2* mutations (52.3%) were much more common than *BRCA1* mutations (9.2%) in our cohort, similar to findings in other studies in the Asian population [34]. Non-*BRCA* genes contributed to 38.5% of the mutation carriers, with *PALB2* (13.8%), *RAD51D* (9.2%), and *ATM* (4.6%) being the majority. *PALB2* is particularly important since lifetime risk for breast cancer can reach 58% in those with family history [51], and NCCN guideline recommends consideration of risk-reducing mastectomy [52]. Among the 8 cases with *RAD51C* and *RAD51D* mutations, 6 (75%) were triple negative breast cancer, in agreement with the recent studies suggesting that mutations in these two genes may confer higher risks of triple-negative or basal subtypes of breast cancer [23, 24]. We also found 2 individuals with protein-truncating mutations in *TP53* and *PMS2* genes, which are high-penetrance cancer predisposing genes and would result in significantly high risk for other cancers. These results showed that testing more than *BRCA1* and *BRCA2* increased the detection rate of clinically actionable high and moderate risk gene mutations, therefore may be an important strategy in the Chinese population. In a study by Thompson et al., significant excess of mutations was only observed for *PALB2* and *TP53* in familial breast cancer cases compared to cancer-free controls [6]. We similarly only found a small number of genes contributing to the majority of mutation carriers. To overcome the challenge of high rates of VUS and questionable clinical actionability, we recommend limiting cancer susceptibility multigene panel in clinical testing to include only a handful of genes with high clinical impact.

Among the six risk factors for hereditary breast cancer in this cohort of all high risk individuals, only bilateral breast cancer showed a statistically significant odds ratio of 3.27 for having a germline mutation in multivariate analysis. In addition, having more risk factors was also associated with a high detection rate of mutations (OR 2.07 for having more than one risk factor). These results suggest that these known risk factors were helpful in identifying individuals for genetic testing and we may need to pay particular attention to those with bilateral breast cancer, even in the absence of family history or young age of onset. Larger cohorts are needed to clarify the significance of ovarian cancer and male breast cancer on breast cancer susceptibility gene mutations in the Asian population. Results from the correlation analysis done with the few high penetrance genes were similar to those done with all the studied genes, suggesting that the correlations were driven by these high penetrance genes including *BRCA1, BRCA2, PALB2,* and *TP53,* which was expected since they represented the majority of the pathogenic variants.

There were some limitations in our study. First, we did not conduct experiments to detect large genome rearrangement (LGR) in all study participants, but used bioinformatics analytical tools to detect copy number variations on the NGS data. This could underestimate the prevalence of LGR in this cohort. However, LGRs have not been shown to contribute significantly to germline mutations in *BRCA* genes in East Asian populations [53, 54]. Second, we were conservative in classifying variants as pathogenic and limited those to protein-truncating variants, which were without ambiguity in assignment of pathogenicity. There were two missense variants classified as likely pathogenic in the ClinVar database, and many missense variants deemed damaging by multiple *in silico* models. However, we did not assign those as pathogenic mutations in this study. We could therefore have underestimated the prevalence of pathogenic mutations. Further studies are underway to evaluate variant segregation with cancer in families, and the accuracy of *in silico* models.

## Conclusions

In this high risk ethnic Chinese cohort, 13.5% had a germline pathogenic mutation in one of twenty breast cancer susceptibility genes, and 8.3% had a *BRCA1*or *BRCA2* mutation. *BRCA* mutation carriers, when diagnosed with breast cancer, were more likely to have lymph node involvement. Their breast cancer specific outcomes were significantly worse even after adjusting for clinical prognostic factors, suggesting *BRCA* mutation to be an independent factor for poor prognosis. Our results highlighted the importance of early testing for breast cancer susceptibility genes, not only for prevention and earlier diagnosis of breast cancer, but also for optimal treatment and surveillance strategies after breast cancer is diagnosed. Further studies are needed to evaluate different treatment approaches for breast cancer in *BRCA* mutation carriers to improve outcome.

## Additional files

Additional file 1: **Figure S1.** Kaplan-Meier estimates of disease-free survival (A), freedom from breast cancer recurrence (B), freedom from distant recurrence of breast cancer (C), and overall survival (D), according to *BRCA1, BRCA2, PALB2* mutation carrier status and all others in the breast cancer cohort. The 5-year and 10-year values are based on Kaplan–Meier estimates of the time to an event. *P*-values are calculated using the log-rank test. (TIFF 520 kb)

Additional file 2: **Table S2.** Effects of *BRCA* germline mutation and other prognostic factors (all covariates) on clinical end points by Cox proportional hazards analysis. The four clinical end points are: breast cancer (BC) recurrence or death, BC recurrence (locoregional or distant), distant recurrence, and death from any cause. Both univariate and multivariate analyses are shown. Statistically significant *P*-values (< 0.05) in the multivariate analyses are shown in bold. (XLSX 14 kb)

Additional file 3: **Table S3.** Correlation between clinical risk factors of hereditary breast cancer and having pathogenic germline mutations in different gene combinations: A. Correlation with all 20 genes; B. Correlation with *BRCA1, BRCA2, PALB2, TP53* only; C. Correlation with *BRCA1, BRCA2* only. (XLSX 14 kb)

Additional file 4: **Table S1.** Carriers of pathogenic mutations with their respective clinical risk factors for hereditary breast cancer, and ClinVar (https://www.ncbi.nlm.nih.gov/clinvar/) accession numbers for novel pathogenic variants. (XLSX 15 kb)

Additional file 5: **Table S4.** Clinical Dataset. Spreadsheet with code-book, containing pathogenic germline gene mutation, hereditary breast cancer risk factors, clinical characteristics, and outcomes in the cohort of breast cancer patients (*N* = 457). (XLSX 65 kb)
