# Supplementary figures and images for: Germline breast cancer susceptibility gene mutations and breast cancer outcomes
Source: BMC Cancer. 2018 Mar 22;18:315. doi: 10.1186/s12885-018-4229-5 (PMC5863855; doi:10.1186/s12885-018-4229-5)

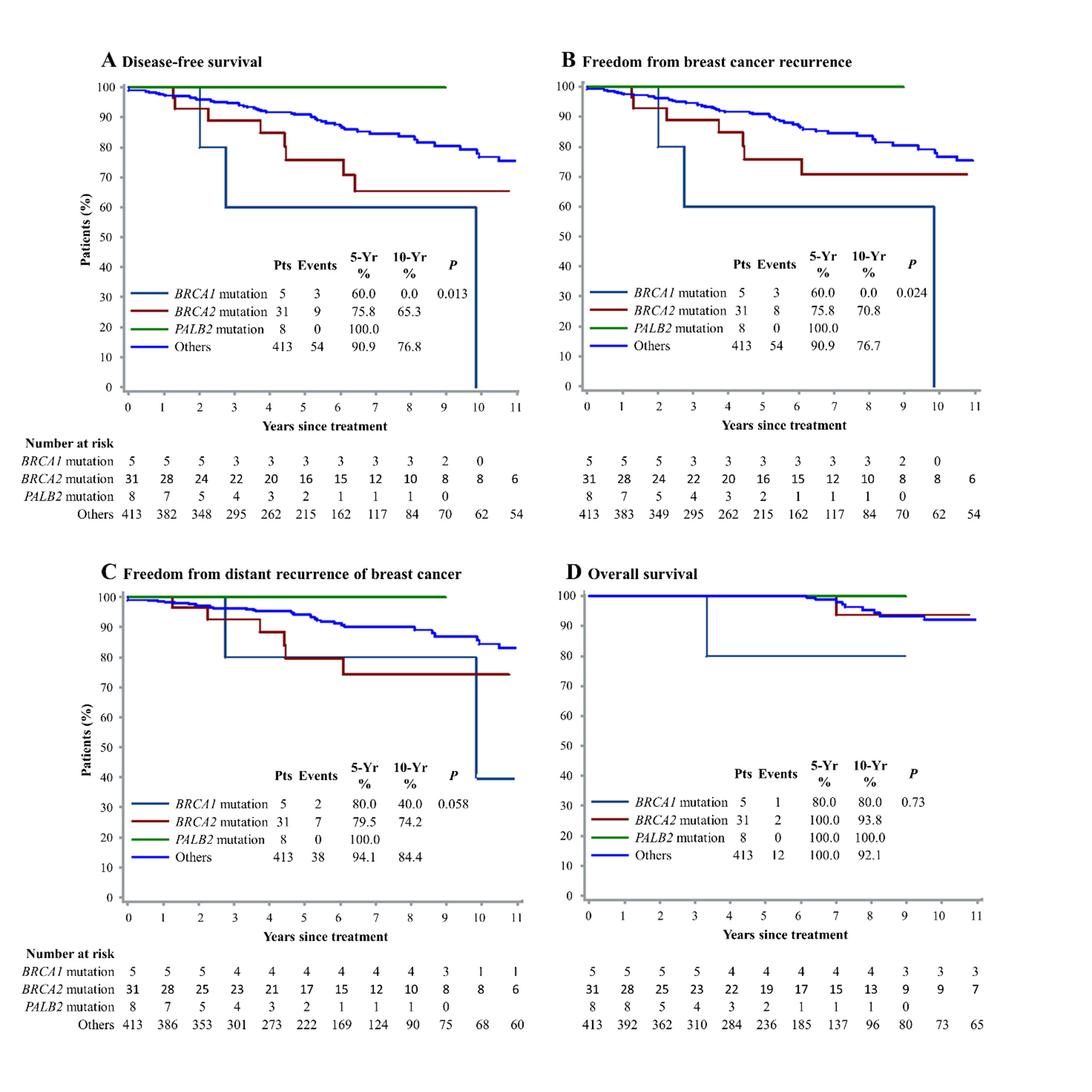

Supplement: Supplementary file 1 — Figure S1. Kaplan-Meier estimates of disease-free survival (A), freedom from breast cancer recurrence (B), freedom from distant recurrence of breast cancer (C), and overall survival (D), according to BRCA1, BRCA2, PALB2 mutation carrier status and all others in the breast cancer cohort. The 5-year and 10-year values are based on Kaplan–Meier estimates of the time to an event. P-values are calculated using the log-rank test. (TIFF 520 kb) [file 12885_2018_4229_MOESM1_ESM.tif]
